# Supplementary material for: Integrating Smoking Cessation Care into a Medically Supervised Injecting Facility Using an Organizational Change Intervention: A Qualitative Study of Staff and Client Views
Source: Int J Environ Res Public Health. 2019 Jun 10;16(11):2050. doi: 10.3390/ijerph16112050 (PMC6603950; doi:10.3390/ijerph16112050)
Supplement: Supplementary file 1 [file ijerph-16-02050-s001.zip › ijerph-519375-Supplementary Files/Supplementary File 1_Manager Interview Guide.pdf]

MANAGER INTERVIEW GUIDE

Document version [4]; dated [28/07/2016]

Hi, my name is Eliza. I am a researcher at the University of Newcastle and a member of the Tackling Nicotine Together research team.

You have been invited today to talk about your personal experiences with the TNT intervention and addressing tobacco smoking as part of Sydney MSIC usual care.

The interview will take approximately 30-60 minutes. I do want to ensure you that your opinions will be very welcome. Although I'll be asking some specific questions to guide our chat today, we can also explore any other aspects of the intervention that you would like to discuss today.

To begin I would like to go over some aspects of the Information statement. Firstly, anything that you say during this chat will remain anonymous. Our conversation today will be audio-taped however no names or identifiers will be used during reporting. If you wish to have any of your comments deleted we can do so at the end of the interview otherwise if you wish to no longer participate at any stage then just let me know and we can stop.

As I mentioned before, there will be some specific questions that I will ask, I understand that it is easy to get caught up on a particular issue but if this happens I will suggest that we move to the next issue to enable sufficient time for all issues to be covered. Do you have any questions that you would like to ask me?

To start I would appreciate it if you could introduce yourself and describe your role and duties at the MSIC.

The TNT project consisted of a number of components. I will introduce each and then ask some specific questions.

1. Organisational engagement

The TNT project aimed to encourage and support your service to address client tobacco smoking

- What made you get involved in the TNT project? Did you believe that the clients utilising your service had a need to quit tobacco smoking?
- Why should the Sydney MSIC address client's tobacco smoking as opposed to any other service that these individuals may be engaged with?
- Did you feel as though the TNT project was kept as a priority in your usual duties and also at staff meetings?

- Was any information provided to the clients prior to the start of the project? If so, how was this done? If not, do you think this would have been beneficial?

2. Identify and support a Champion

The support champion aimed to ensure that client tobacco smoking was addressed and treated at the Sydney MSIC.

- Was it your role to decide who the support champion was? If so, what influenced your decision?
- Were there any changes made to this person's current role at the service to ensure that client tobacco smoking was assessed and treated? What were these?
- Do you think that having someone identified in this role kept tobacco smoking on the agenda at the MSIC?
- Do you think what has been achieved at the MSIC in terms of client tobacco smoking would have been achieved without a service-specific champion?
- Do you feel as though there is a group effort in driving the importance of addressing client tobacco smoking at the MSIC or is it a sole person.

If a sole person- What do you think would happen if they left the service?

- Do you believe the service will continue to address client tobacco smoking?
  - ***If yes,*** What processes have been implemented to ensure this?
- Are there ways you think this could be an ongoing or identified role? How do you think this could continue?

3. Tobacco Policy Promotion

What is the current policy on tobacco smoking at MSIC?

- Has this policy changed in the past year? Did participating in the TNT project cause you to re-examine your smoking policies and tobacco smoking protocols?

If yes, in what way?

- Do you think the staff are well informed about the tobacco policy? Including the external premises (entry & exit)

  If no, how could this be improved?

  If yes, how was this done? Meetings, signs etc…

- Is the policy enforced? How?
- Were there any barriers to addressing tobacco smoking on site at the MSIC?
- Do you think the tobacco policy is viewed as a priority/important by the service and its staff?

4. Smoker ID System

Do clients of the Sydney MSIC have their smoking status assessed?

How?

- Is this a good system? Does it prompt further action or care by staff?
- Were you involved in trialling or making suggestions to the system?

  If yes, what were these specifically?

- Did you provide information to your staff about the changes prior to the system being implemented? How did you do this and do you believe this helped?
- Do you believe this helped staff to change their practices and address client tobacco smoking?

5. Education & Resources (Training)

Have you participated in any formal tobacco or smoking cessation training in the past year?

- Was this helpful? Was it acceptable?
- Was enough information provided?
- Do you believe that your staff integrated what was learnt into practice? How so?

6. Include Evidence Based Tobacco Treatments (NRT)

The TNT team provided a range of Nicotine Replacement Therapies to be provided to clients of the MSIC

- Do you think the service is well placed to provide Nicotine Replacement Therapy?
- Was there any changes made to the current system of care to ensure that NRT was provided?

  If yes, how did this occur? Do you think this assisted staff?

- Do you think this could be an ongoing part of what the MSIC does?

7. The last section aims to examine a broad overview of your experiences with the TNT project as a whole

- Overall, when you think about your experiences with the TNT project, what do you think have been the main facilitators for encouraging client tobacco smoking to be addressed?
- Overall, when you think about your experiences with the TNT project, what do you think have been the main barriers to encouraging client tobacco smoking at the MSIC?

Conclusion (when approximately 5 minutes are remaining)

So to summarise the main points from our chat today

1.

2.

3.

If you would like to add anything else for consideration this is the time to do so.

Thank you for agreeing to be interviewed today, I will turn the tape off now.
