# Supplementary material for: Integrating Smoking Cessation Care into a Medically Supervised Injecting Facility Using an Organizational Change Intervention: A Qualitative Study of Staff and Client Views
Source: Int J Environ Res Public Health. 2019 Jun 10;16(11):2050. doi: 10.3390/ijerph16112050 (PMC6603950; doi:10.3390/ijerph16112050)
Supplement: Supplementary file 1 [file ijerph-16-02050-s001.zip › ijerph-519375-Supplementary Files/Supplementary File 2_Staff Interview Guide.pdf]

STAFF INTERVIEW GUIDE

Document version [4]; dated [28/07/2016]

Hi, my name is Eliza. I am a researcher at the University of Newcastle and a member of the Tackling Nicotine Together research team.

You have been invited today to talk about your personal experiences with the TNT intervention and addressing tobacco smoking as part of Sydney MSIC usual care.

The interview will take approximately 30-60 minutes. I do want to ensure you that your opinions will be very welcome. Although I'll be asking some specific questions to guide our chat today, we can also explore any other aspects of the intervention that you would like to discuss today.

To begin I would like to go over some aspects of the Information statement. Firstly, anything that you say during this chat will remain confidential. Our conversation today will be audio-taped however no names or identifiers will be used during reporting. If you wish to have any of your comments deleted we can do so at the end of the interview otherwise if you wish to no longer participate at any stage then just let me know and we can stop.

As I mentioned before, there will be some specific questions that I will ask, I understand that it is easy to get caught up on a particular issue but if this happens I will suggest that we move to the next issue to enable sufficient time for all issues to be covered. Do you have any questions that you would like to ask me?

To start I would appreciate it if you could introduce yourself and describe your role and duties at the MSIC.

The TNT project consisted of a number of components. I will introduce each and then ask some specific questions.

1. Organisational engagement

The TNT project aimed to encourage and support your service to address client tobacco smoking

- How were you told about the TNT project and MSIC's involvement?
- Were you involved in getting ready for this project to commence? Or was there any noticeable changes in your duties or duties of others?
- When you were told about the MSIC participating in the TNT project did you have any concerns? Were these addressed by management or the project team?
- Did you feel as though you had the support of the MSIC's management team to implement tobacco smoking care?

- Do you think support increased as a result of this project?
- Was getting that organisational leadership support an important factor for you to be able to address client tobacco smoking?

2. Identify and support a Champion

The support champion aimed to ensure that client tobacco smoking was addressed and treated at the Sydney MSIC.

- Do you think that having someone in this role kept tobacco smoking on the agenda at the MSIC?
- Do you think that by having a champion, it made it easier to address tobacco smoking at the MSIC?
- Do you think what has been achieved at the MSIC in terms of client tobacco smoking would have been achieved without a service- specific champion?
- Did you feel that the champion was influential in ensuring that client tobacco smoking was addressed and treated?
- Do you feel as though there is a group effort in driving the importance of addressing client tobacco smoking at the MSIC or is it a sole person?

<u>If a sole person-</u> What do you think would happen if they left the service?

- Are there ways you think this could be an ongoing or identified role? How do you think this could continue?

3. Tobacco Policy

What is the current policy on tobacco smoking at MSIC?

- Has this policy changed in the past year? Did participating in the TNT project cause you to re-examine your smoking policies and tobacco smoking protocols?

If yes, in what way?

- Do you think the staff are well informed about the tobacco policy? Including the external premises (entry & exit)

    If no, how could this be improved?

    If yes, how was this done? Meetings, signs etc.…
- Is the policy enforced? How?
- Were there any barriers to addressing the onsite smoking of individuals on MSIC grounds?
- Do you think the tobacco policy is viewed as a priority/important by the service and its staff?

4. Smoker ID System

Do clients of the Sydney MSIC have their smoking status assessed more or less after the TNT project? How?

- Is this a useful system? In what way is it good or bad?
- How does this system work?
- Were you involved in trialling or making suggestions to this system?

    If yes, what were these specifically? Did this help with ease of screening or functionality with current practices?
- Do you feel that it aids you to be able to address client tobacco smoking?
- Any concerns?

5. Education & Resources (Training)

Have you participated in any formal tobacco or smoking cessation training in the past year?

- Was this helpful? Was it acceptable?
- Was enough information provided? How did it make you feel about addressing tobacco smoking?
- Do you think that you integrated what you learnt into practice? How so?

6. Include Evidence Based Tobacco Treatments (NRT)

Have you ever provided NRT such as patches, lozenges, inhalators to MSIC clients?

- How did that go?
- What would you do? Did you create this process or was this consistent with what other staff were doing?
- Did you feel comfortable/ prepared/ well-informed to do this?
- Do you think the service is well placed to provide NRT?
- Do you think this could be an on-going part of what MSIC does?
- Did you create a record of what you provided to patients? Do you think this helped you provide the NRT to clients?

7. The last section aims to examine a broad overview of your experiences with the TNT project as a whole

- Overall, when you think about your experiences with the TNT project, what do you think have been the main facilitators for encouraging client tobacco smoking to be addressed?
- Overall, when you think about your experiences with the TNT project, what do you think have been the main barriers to encouraging client tobacco smoking at the MSIC?

Conclusion (when approximately 5 minutes are remaining)

So to summarise the main points from our chat today

1.

2.

3.

If you would like to add anything else for consideration this is the time to do so.

Thank you for agreeing to be interviewed today, I will turn the tape off now.
