# Supplementary material for: Integrating Smoking Cessation Care into a Medically Supervised Injecting Facility Using an Organizational Change Intervention: A Qualitative Study of Staff and Client Views
Source: Int J Environ Res Public Health. 2019 Jun 10;16(11):2050. doi: 10.3390/ijerph16112050 (PMC6603950; doi:10.3390/ijerph16112050)
Supplement: Supplementary file 1 [file ijerph-16-02050-s001.zip › ijerph-519375-Supplementary Files/Supplementary File 3_Client Discussion Guide.pdf]

**CLIENT FOCUS GROUP DISCUSSION GUIDE**

**Document version [4]; dated [28/07/2016]**

**Introduction**

Hi everyone. My name is ELIZA and this is ASHLEIGH we are from the University of Newcastle and will be running today's discussion.

Today we are here to talk about your tobacco smoking being addressed at the Sydney MSIC. The discussion will take about 45 minutes and we welcome everyone's opinion. I will be asking some specific questions today but you can talk about any other aspects you want of having your tobacco smoking addressed by staff at the Sydney MSIC.

To begin I would like to go over some aspects of the information statement that you were provided on entry to the room today. Our discussion today will be audio-taped however no names or any other identifying information will be used when reporting the results of this discussion. If you wish to have any of your comments deleted we can do so at the end of the discussion otherwise if you wish to no longer participate at any stage then just let me know and we can stop.

As I mentioned before, there will be some specific questions that I will ask and sometimes it is easy to get caught up on a particular issue but if this happens I will ask that we move on. It would be good if we try not to talk over each other, and of course it goes without saying that we'll always respect each other's views. Do you have any questions that you would like to ask us?

**1. Introduction**

The first thing I want to do is for everybody to say something about their smoking- maybe how long you've been smoking for, if you enjoy it or what you don't like so much about it- something to start us off- starting on my left.

**2. How is tobacco smoking assessed at MSIC?**

Have you been asked by staff at the Sydney MSIC whether you smoke tobacco?

- When did this happen? On entry to the service?
- How did you feel about it- did it fit with the kind of care you already receive at the MSIC?
- Is it just like being asked about other substance use?
- Is this something that the service should do?

*If yes,* who do you think is the best person or people to talk to you about your smoking here at the MSIC?

*If not,* what other service should ask you about your tobacco smoking or where would you go to get help with your smoking? A Doctor (GP)?

**3. How is tobacco smoking treated at the MSIC- the strategies**

Have you received advice to quit or been asked if you would like help to quit tobacco smoking from staff at MSIC?

- Has this attention to your smoking been any different lately, say in the last year?
- Was this from a particular person at the MSIC or have a number of staff spoken to you?
- Did it motivate you/make you think about quitting or decreasing your tobacco smoking? Why/ Why not?

Have you been offered referral to individual or group counselling by MSIC staff?

- Was this a private or group counselling session that focused on tobacco smoking?

  *If yes,* did you go? What did/ did you not like about it?

- If a counselling session was dedicated to your tobacco smoking would you like to attend this type of group and should this group be conducted by staff at the Sydney MSIC?
- What about Quitline, the telephone counselling service, did MSIC staff offer for someone from the quitline to call you to talk about your tobacco smoking?

*If yes,* did you receive a call? What did you like/ not like about it?

*If no,* would you have liked to use this service? Why/ Why not?

Have you ever been offered any NRT like patches or gum from MSIC staff?

- Did you think that being provided with free NRT at the MSIC was appropriate or should this be provided by another service?
- How did this happen? Did you ask for this or did the staff member just provide this to you?
- Did you use it / like it? Why/why not?

**4. The tobacco smoking policy at the MSIC**

As you may already be aware the MSIC has a no smoking policy inside the service and more recently tobacco smoking was banned at the entrance and exit of the building

- Has this changed the way that you use the service i.e. you need to have a smoke in a different area before coming to the MSIC?
- Do you think this is appropriate? Has it changed your views on using the Sydney MSIC?

**5. This last section is dedicated to your thoughts and feeling regarding tobacco smoking at the Sydney MSIC**

Overall do you think that MSIC is a good place to receive help with your tobacco smoking?

- Why/ why not? if not, where would be a good place to get quitting help?
- Is there anything else that you would like to add that has not been already covered in our discussion today?

**Conclusion**

That concludes the main aspects of the focus group today. On behalf of both of us we would like to express how appreciative we are of you sharing your thoughts and experiences. If there was anything anyone would like to add or clarify you may like to see us now before we end our final discussions.

Thank you again for all your comments; I will turn the tape off now.
